# Incidentally identified ductus arteriosus aneurysm in eight adults: a case series

**DOI:** 10.1259/bjrcr.20200097

**Published:** 2021-03-18

**Authors:** Mayo Yukimoto, Tomohisa Okuma, Etsuji Sohgawa, Mariko M Nakano, Taro Shimono, Yukio Miki

**Affiliations:** 1 Department of Diagnostic and Interventional Radiology, Graduate School of Medicine, Osaka City University, Osaka, Japan 1-4-3 Asahimachi, Abenoku, Osaka, Japan 545-8585; 2 Department of Diagnostic Radiology, Osaka City General Hospital, Osaka Japan 2-13-22 Miyakojima-hondori, Miyakojima-ku Osaka, Japan 534-0021; 3 Department of Radiology, National Hospital Organization Osaka Minami Medical Center, Kawachinagano, Japan 2-1 Kidohigashi-cho, Kawachinagano, Osaka, Japan 586-8521

## Abstract

Ductus arteriosus aneurysm (DAA) in adulthood is a rare entity. We retrospectively reviewed our medical records from the past 10 years and identified 8 cases of adult DAA (6 males and 2 females aged between 69 and 89 years; mean, 76 years), using multiplanar reconstruction and three-dimensional reconstruction CT images. The aneurysm was suspected incidentally in all cases based on the results of chest radiographic screening or post-operative follow-up CT for lung or colon cancer. All eight patients were asymptomatic but had a history of or concurrent hypertension (*n* = 5, 62.5%), diabetes mellitus (*n* = 3, 37.5%), cerebrovascular disease (*n* = 3, 37.5%), ischemic heart disease (*n* = 1, 12.5%), and cardiac failure (*n* = 1). All patients had no history of trauma (*n* = 8, 100%). Six had a history of cigarette smoking. The aneurysm size ranged from 2.0 × 4.0 to 6.3 × 5.3 cm (mean, 3 × 5 cm). The surgical procedures used were four cases of total arch replacement and two cases of thoracic endovascular aortic repair. Two patients were not surgically treated. The median follow-up was 14.5 months (range, 2 months to 9 years). In the two patients who were not surgically treated, the aneurysm enlarged in one, and remained unchanged in the other. Of the six surgically managed cases, one was lost to follow-up, and another patient died of an unrelated cause. The remaining four cases had no enlargement of the aneurysm. No ruptures were reported in any of the cases. DAA should be considered when a saccular aneurysm is located in the minor curvature of the aortic arch and extending toward the left pulmonary trunk in adult patients. Differentiating adult DAA is important, because it is associated with a high risk of rupture due to the fragile nature of true aneurysms.

## Introduction

At birth, the physiological closure of the ductus arteriosus starts at the pulmonary artery end and progresses toward the aortic end. Ductus arteriosus aneurysm (DAA) is believed to occur by progressive dilatation of the ductus diverticulum, which forms as a result of delayed or incomplete closure of the aortic side of the duct.^1,2^ Anatomically, DAA is a form of saccular aortic aneurysm that extends from the aortic arch toward the pulmonary artery. Rarely exceeding 2 cm in diameter, DAA has been detected at 8.8% of incidence during newborn screening.^3,4^ While spontaneous regression is reported in about 70% of neonates, the other 30% may develop thrombi progressively.^5^

The adult form of DAA is even rarer, and only a handful of cases have been reported.^1,2,6–12^ Early intervention is a key, as potentially fatal complications associated with true aneurysm, such as compression of surrounding structures, rupture of the weakened vessel wall, thromboembolism, and infection, may occur.^1,7,8^ However, diagnostic imaging studies of adult DAA are limited,^6,9–12^ and some DAAs appear to have been misdiagnosed as saccular, true aneurysms of the aortic arch.^13,14^ The purpose of the present retrospective study was to identify and review important imaging features of adult DAA in light of previous reports.

## Study protocol

The study was approved by the institutional ethics committee (Approval No. 4388). Electronic medical records between April 1, 2010 and September 30, 2019 were searched for possible DAA patients, and patient information (*i.e.* gender, age, previous and concurrent illness such as diabetes, hypertension, cardiovascular disease, and cerebrovascular disease, history of cigarette use, and reason for hospital visit), as well as CT, angiography, ultrasonography, and surgical data (*i.e.* follow-up period, aneurysm size, thrombus formation, calcification, ligamentum arteriosum connection, and surgical procedure) were extracted.

According to Danza et al^10^ and using multiplanar reconstruction and three-dimensional (3D) reconstruction contrast CT images, a diagnosis of DAA was made based on the demonstration of: (1) a sac-like structure, which was (2) extending from the ductus arteriosus toward the left pulmonary trunk, and the absence of (3) past trauma (evidence of trauma on CT), (4) arterial dissection, and (5) arteritis-based on the consensus of two radiologists with 15 and 20 years of experience.

Dynamic contrast-enhanced CT was used in seven cases, and single-phase contrast-enhanced CT in one case. For dynamic CT, a scan was performed 20–30 sec and 120 sec after intravenous contrast injection. Contrast-enhanced CT scanning was performed 60 sec after intravenous contrast injection during the equilibrium phase. The slice thickness was 1 mm and 0.625 mm in two and five of seven cases of dynamic CT, respectively, and 5 mm in single-phase CT.

## Results

The results are summarized in Table 1. DAA was confirmed in a total of eight cases, two females and six males (mean age, 76 years; median age, 78 years). The median follow-up period was 14.5 months (range, 2 months to 9 years). Complications of DAA such as symptoms associated with the compression of surrounding thoracic structures, rupture, and infection were not reported in any of the patients. In all eight cases, the aneurysm was asymptomatic and suspected incidentally by plain chest X-ray or follow-up CT. Other clinically relevant information included concurrent hypertension (*n* = 5, 62.5%), concurrent diabetes mellitus (*n* = 3, 37.5%), history of cerebral infarction (*n* = 2, 25.0%), and history of cigarette smoking (*n* = 6, 75.0%).

**Table 1.** Summary of eight patients with ductus arteriosus aneurysm

Case	Age (y)/Gender	Reason for referral	LA connection	Aneurysm size (cm)	Follow-up period	Outcome	Surgery	Hypertension	Diabetes mellitus	Cerebrovascular condition	Calcification	Thrombus formation	Smoking habit	History
1	78M	Incidentally found by X-ray	Yes	3.5 × 5.4	4 m	Unchanged	ABV, open stent	Yes	Yes	No	Yes	Yes	40/day × 53 y	After lung cancer operation
2	72F	Incidentally found by CT	Yes	2.0 × 4.0	1 y 9 m	Unchanged	ABV, open stent	Yes	No	Cerebral infarction	Yes	Yes	Yes (details unknown)	AAA, Aortic valve insufficiency
3	89M	Incidentally found by X-ray	No	4.4 × 5.9	17 m	Enlarged (7.0 × 5.3)	None	Yes	No	No	Yes	Yes	None	Hepatitis B, aortic dissection
4	72M	Incidentally found by CT	Yes	3.2 × 4.8	3 y	Unchanged	ABV	Yes	No	No	Yes	Yes	40/day × 50 y	
5	81M	Suspected by routine X-ray check	Yes	5.0 × 2.8	9 y	Unchanged	TEVAR	No	Yes	No	No	Yes	None	Angina
6	73M	Incidentally found by CT	No	6.3 × 5.3	1 y	Death	ABV, open stent	No	Yes	Subarachnoid hemorrhage	Yes	Yes	10/day × 30 y	
7	69M	Incidentally found by CT	Yes	3.0 × 5.9	5 m	Unchanged	None	Yes	No	No	Yes	Yes	5/day × 35 y	After lung cancer operation
8	81F	Incidentally found by X-ray	No	5.7 × 4.0	2 m	Lost to follow-up	TEVAR	No	No	Cerebral infarction	Yes	Yes	10/day × 45 y	AAA

AAA, abdominal aortic aneurysm; ABV, artificial blood vessel; CT, computed tomography; LA, ligamentum arteriosum; TEVAR, thoracic endovascular aortic repair.

The mean aneurysm size was 4.0 × 5.1 cm (ranging from 2.0 × 4.0 to 6.3 × 5.3 cm). Among all eight cases, intraluminal calcification and thrombus formation were found in seven (87.5%) and 100% of the patients, respectively. The ligamentum arteriosum was identified in five (62.5%) of the patients and was connected to the DAA. All patients had no history of trauma (*n* = 8, 100%). Two cases were evaluated by ultrasonography to assess cardiac function. After the treatment, ultrasound examination performed for follow-up showed no abnormalities in both of them. Six patients had angiography for artificial blood vessel replacement, but none had angiography for diagnosis.

Surgical intervention included total arch replacement in four (50.0%) and thoracic endovascular aortic repair in one (25.0%). Two patients were untreated. In these two cases, the aneurysm enlarged in one and remained unchanged in the other. Of the six surgically treated cases, one was lost to follow-up and another died from subarachnoid hemorrhage. No DAA-related deaths were recorded. The post-surgical course was uneventful in the remaining four cases with no evidence of aneurysm enlargement.

## Representative cases

### Case 1

**Figure 1.**
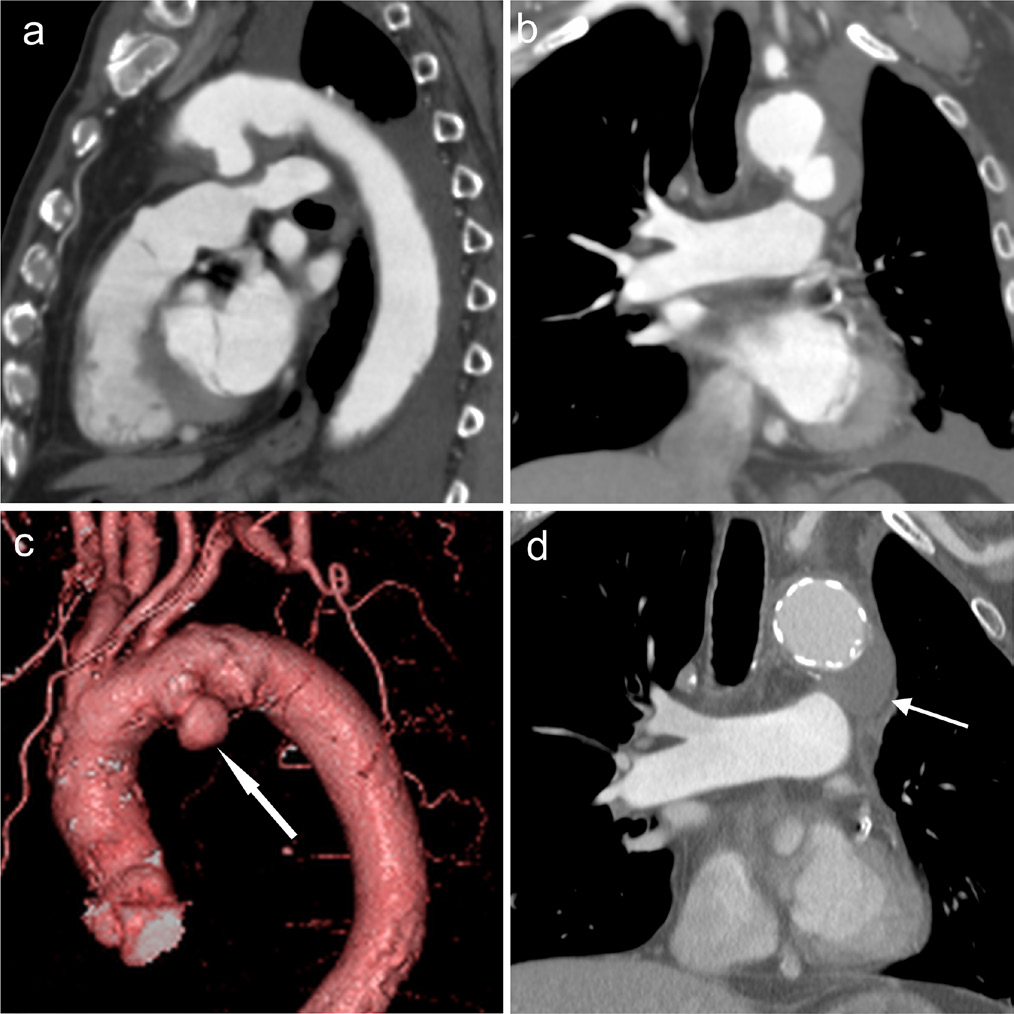
Ductus arteriosus aneurysm (white arrows) shows extending posteroinferiorly from the left side-of the aortic arch on (**a**) sagittal multiplanar reconstruction, (**b**) coronal multiplanar reconstruction, and (**c**) 3DCT angiography images in Case 1. (**d**) After complete arch replacement and open stent grafting, dilated aneurysms diminished, resulting in a good outcome during follow-up.

A 78-year-old male was referred to our hospital (Osaka City University hospital) for a contrast CT study for possible thoracic aortic aneurysm suspected by plain chest radiography. He was medically managed for diabetes mellitus and hypertension and had a history of pulmonary carcinoma in the left upper lobe, which had been surgically treated. He had a history of cigarette smoking (Brinkman index, 2130). A saccular aneurysm (3.5 × 5.4 cm) extended posteroinferiorly to the left from the aortic arch (Figure 1(a)(b)(c), arrows). Mural thrombosis was present. Total arch replacement and open stent grafting were successful, and dilatation of the aneurysm did not recur (Figure 1(d)).

### Case 2

**Figure 2.**
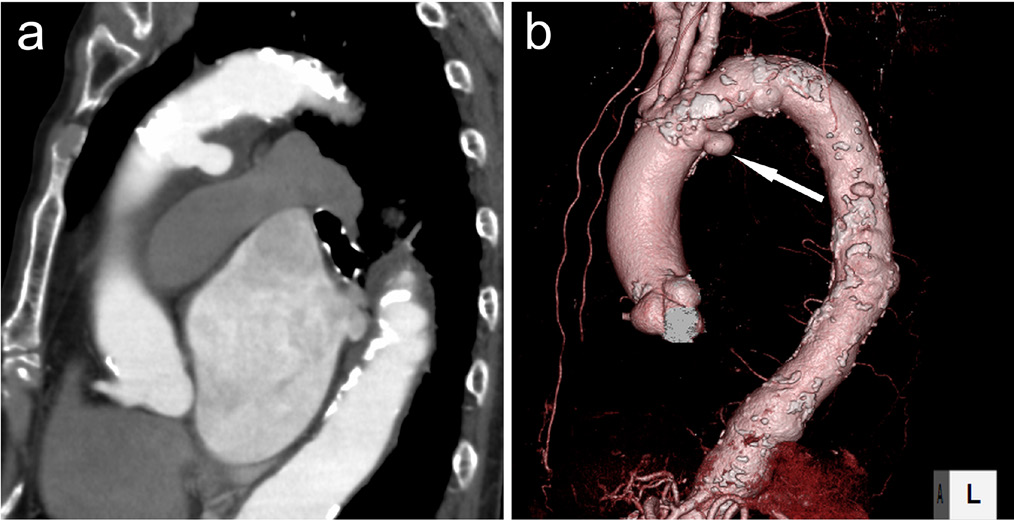
Ductus arteriosus aneurysm (white arrows) extending posteroinferiorly from the left side of the aortic arch on (**a**) sagittal multiplanar reconstruction and (**b**) 3DCT angiography images in Case 2. 3D, three-dimensional.

A 72-year-old female with a history of abdominal aortic aneurysm was referred to our hospital for possible thoracic aortic aneurysm suspected by follow-up contrast-enhanced CT. Smoking history was unknown. DAA was observed as a saccular structure of 2.0 × 4.0 cm extending posteroinferiorly from the left side of the aortic arch (Figure 2(a)(b), arrows). The clinical course after total arch replacement and open stent grafting was uneventful without regrowth of the DAA.

### Case 3

**Figure 3.**
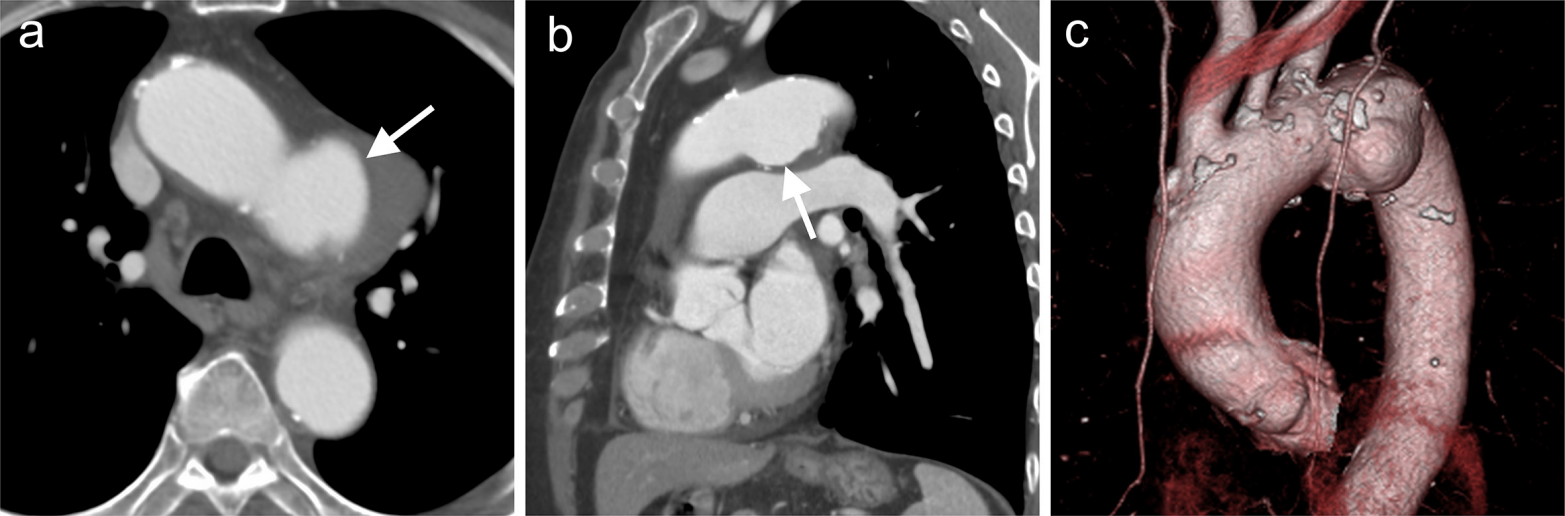
Ductus arteriosus aneurysm (white arrows) shown on is reveal in (**a**) axial image, and (**b, c**) 3DCT angiography imaging in Case 3. 3D, three-dimensional.

An 89-year-old male with medically managed hypertension was referred for a contrast CT study for possible thoracic aortic aneurysm suspected by plain chest radiographs. He had no smoking history. DAA was observed as a sac-like structure of 4.4 × 5.3 cm extending inferiorly from the left side-of the aortic arch (Figure 3(a)(b)(c), arrows) with a mural thrombus. The patient was untreated, and enlargement of the DAA to 7.0 × 5.3 cm was confirmed after 1.5 years (image not shown).

## Discussion

Although 8 cases in 10 years are rare, it is important to differentiate adult DAA by diagnostic imaging. Its natural course is generally considered grim, given the very fragile nature of the aneurysmal wall, which is constantly at risk of rupture, and premature death may result without intervention.^8,15,16^ In our study, six patients were surgically treated, and no ruptures were reported. The DAA was suspected incidentally in all cases based on the results of radiographic imaging. Previously reported imaging features of DAA include a saccular structure originating from the ductus arteriosus toward the left pulmonary trunk and “triple star” alignment of the ascending aorta, aneurysmal sac, and descending aorta on the same 3DCT slice beneath the aortic arch.^17^ Compared to atherosclerotic aneurysms in the distal aortic arch, DAA is located in the minor curvature of the distal aortic arch at the bifurcation of the left subclavian artery, and its end is directed toward the upper left subclavian artery.^17^ The attachment of the ligamentum arteriosum has been reported to be useful for diagnosis,^18^ but only observed in one-third of the cases according to another study.^1^ In our study, a diagnosis of DAA was made when all of the five criteria, described in the STUDY PROTOCOL section, were met. The “triple star” alignment was observed in 5/8 patients (62.5%), and the ligamentum arteriosum was identified in 5/8 (62.5%). It was difficult to detect the ligamentum arteriosum in some cases due to the size and inferior dilatation of the DAA. Both the “triple star” alignment and the ligamentum arteriosum were observed in 3/8 patients (37.5%). The “triple star” alignment and the ligamentum arteriosum are easy-to-recognize image features and were found in our study at a higher frequency than in the literature.

3DCT angiography is useful to evaluate the DAA. The exact relationship of a ductal aneurysm with the aorta and pulmonary artery can be understood from CT images. With multiplanar reconstruction, we can easily identify the spatial location of the aneurysm.^19^ Angiography was used to diagnose aneurysms, but nowadays, it is also used to evaluate the position of the lesion in relation to the aortic arch for treatment planning, and to measure the ends, shape, blood flow, and size of the DAA.^10,12^ Few papers have evaluated the DAA by ultrasonography alone. Ultrasonographic evaluation is challenging depending on the conditions such as interference by the lung parenchyma and the limited acoustic window of adult patients.^12,20^ Imaging studies such as echocardiography and coronary angiography are most likely done pre-operatively to rule out existing conditions.

Although the exact mechanism of DAA formation remains unclear, the most frequently cited theory is that the incomplete closure of the aortic end of the ductus arteriosus followed by gradual enlargement due to the systolic blood pressure. The local fragility of the aortic wall, reflecting the structural changes during ductal closure, may also play a part.^2,8–10,12,20,21^ DAA occurs from the anterior wall of the aorta and is histologically characterized by reduced elastic fibers in the aneurysmal wall and mucoid degeneration of the media.^22^ In this study, the proportion of patients with calcification, hypertension, and history of smoking was high. In addition, all patients were advanced in age. It is possible that slow but progressive arteriosclerotic changes due to advanced age, smoking, gender effect, and hypertension resulted in gradual dilatation of the diverticulum over a long period of time.^1,10,15^ In addition to congenital factors, these acquired factors may contribute to the formation of DAA.

Post-traumatic aneurysm is an important differential diagnosis of the aneurysms located in the aortic arch. Post-traumatic aneurysms have two mechanisms. A generally accepted mechanism is blunt trauma resulting in rapid deceleration. The other proposed mechanism is an anteroposterior compression force resulting in posteroinferior displacement of the manubrium, first rib, and medial clavicle, which impinge on the aorta and compress it against the thoracic spine posteriorly.^23^ Chronic traumatic aortic aneurysms can gradually grow and cause a rupture like DAA and compression of the laryngeal nerve and esophagus several years after the injury.^24^ Thus, confirmation of the presence or absence of past trauma is essential when differentiating from DAA, but none of our cases had a history of trauma.

In our study, all patients were asymptomatic, and an aneurysm was suspected incidentally as a result of a regular health checkup or during follow-up for other conditions. Pontone et al showed DAA can be found incidentally by chest CT.^20^ Pastuszko et al reported that hoarseness of voice resulting from compression of the laryngeal nerve is a common sign of adult DAA.^15^ Dyspnea, cough, chest pain, and dysphagia due to mechanical compression as well as bloody sputum and hemoptysis may also occur and are possible signs of rupture into the airway and esophagus.^7,16^

In our eight cases, six were surgically treated (four cases by total arch replacement and two cases by thoracic intravascular aortic repair) with successful results without complications or worsening of the condition. Early surgical intervention is recommended for DAAs greater than 3 cm based on the statistically higher risk of rupture. Because the surgical risk is also higher in older patients, endovascular stent grafting has been used and resulted in successful outcomes in recent years.^25,26^ One of our six patients who received surgical treatment died of an unrelated cause (subarachnoid hemorrhage), while the remaining four did not show any signs of aneurysm growth by follow-up CT. Surgical intervention was post-poned in two patients (25.0%) to prioritize the treatment of other diseases, and the aneurysm expansion was confirmed in one patient.

## Learning points

1. Although, adult DAA is rare, knowledge of these imaging features of DAA may be helpful for management decisions.
2. DAA should be suspected when a saccular aneurysm is located in the minor curvature of the aortic arch and extends toward the left pulmonary trunk.
3. Asymptomatic, incidentally diagnosed patients have a low risk of complications from intervention with a good outcome.

## Consent

Due to the retrospective nature of the study, it was not possible to obtain informed consent from the patients. Instead, we used the opt-out method by publicly releasing and delivering the study information to the patients, and patient anonymity has been maintained.
